# Miya Improves Osteoarthritis Characteristics *via* the Gut-Muscle-Joint Axis According to Multi-Omics Analyses

**DOI:** 10.3389/fphar.2022.816891

**Published:** 2022-05-20

**Authors:** Tianyang Xu, Dong Yang, Kaiyuan Liu, Qiuming Gao, Zhongchen Liu, Guodong Li

**Affiliations:** ^1^ Department of Orthopedics, Shanghai Tenth People’s Hospital, School of Medicine, Tongji University, Shanghai, China; ^2^ Department of General Surgery, Shanghai Tenth People’s Hospital, School of Medicine, Tongji University, Shanghai, China

**Keywords:** Miya, osteoarthritis, gut microbiota, metabolites, gut-muscle-joint axis

## Abstract

**Background:** The gut microbiota is associated with osteoarthritis (OA) progression. Miya (MY) is a product made from *Clostridium butyricum*, a member of gut microbiota. This study was conducted to investigate the effects of MY on OA and its underlying mechanisms.

**Methods:** An OA rat model was established, and MY was used to treat the rats for 4 weeks. Knee joint samples from the rats were stained with hematoxylin-eosin, and fecal samples from the OA and OA+MY groups were subjected to 16S rDNA sequencing and metabolomic analysis. The contents of succinate dehydrogenase and muscle glycogen in the tibia muscle were determined, and related genes and proteins were detected using quantitative reverse transcription polymerase chain reaction and western blotting.

**Results:** Hematoxylin and eosin staining showed that treatment with MY alleviated the symptoms of OA. According to the sequencing results, MY significantly increased the Chao1, Shannon, and Pielou evenness values compared to those in the untreated group. At the genus level, the abundances of *Prevotella*, *Ruminococcus*, *Desulfovibrio*, *Shigella*, *Helicobacter*, and *Streptococcus* were higher in the OA group, whereas *Lactobacillus*, *Oscillospira*, *Clostridium*, and *Coprococcus* were enriched after MY treatment. Metabolomic analysis revealed 395 differentially expressed metabolites. Additionally, MY treatment significantly increased the succinate dehydrogenase and muscle glycogen contents in the muscle caused by OA (*p* > 0.05). Finally, *AMPK*, *Tfam*, *Myod*, *Ldh*, *Chrna1*, *Chrnd*, *Rapsyn*, and *Agrin* were significantly downregulated in the muscles of OA mice, whereas *Lcad*, *Mcad*, and *IL-1β* were upregulated; MY significantly reversed these trends induced by OA.

**Conclusions:** MY may promote the repair of joint damage and protect against OA via the gut-muscle-joint axis.

## Introduction

Osteoarthritis (OA) is characterized by degeneration of the articular cartilage and subchondral bone, often leading to pain, joint stiffness, and disability (Thomas et al., 2017). The pathogenesis of OA is complex and is thought to result from interactions among age, genetics, metabolism, injury, obesity, and inflammatory mechanisms (Vina and Kwoh, 2018). Approximately 30.8 million adults in the United States and 300 million people worldwide have OA (Abramoff and Caldera, 2020), creating an important economic burden in terms of medical care costs, lost wages, and lost economic productivity (Zhao et al., 2019). There are currently no approved treatments for OA, with treatments aimed only at symptom relief, in addition to slowing/stopping OA progression (Ghouri and Conaghan, 2019). Recently, analgesia, including paracetamol, topical and oral non-steroidal anti-inflammatory drugs, opioids, and corticosteroids, remain the main drug-based method for relieving OA (Ghouri and Conaghan, 2019; Kloppenburg and Berenbaum, 2020). However, the benefits of these drugs are limited, and their long-term use can lead to various side effects (Kloppenburg and Berenbaum, 2020). Therefore, novel drugs and targets for managing OA are urgently needed.

Previous studies suggested that the gut microbiota (GM) is associated with OA progression, and GM dysfunction contributes to promoting metabolic syndrome and chronic low-grade inflammation, thus leading to musculoskeletal injury and weakness (Biver et al., 2019; Kloppenburg and Berenbaum, 2020). The GM, consisting of more than 10^14^ bacteria and over 1,000 species as well as fungi, viruses, phages, parasites, and archaea, colonizes the entire gastrointestinal tract in a variable manner and represents a true ecosystem (Schoeler and Caesar, 2019). The GM exerts a variety of functions, including the maintenance of metabolic homeostasis, protection from infection, absorption of nutrients, and development of systemic and mucosal immunity (Kamada et al., 2013; De Sire et al., 2018). In healthy individuals, GM homeostasis is maintained by controlling the growth of pathogenic microorganisms. Once the balance is broken, known as dysbiosis, pathogenic microbes flourish, leading to intestine-related diseases such as allergic disease, obesity, colorectal cancer, inflammatory bowel disease, diabetes, autism, and cardiovascular disease (Wang and Zhao, 2018). A previous study of a rat OA model indicated that the altered abundance of GM *Lactobacillus* spp. and *Methanobrevibacter* spp. was related to the Mankin scores of articular cartilage injury (Collins et al., 2015). Another study showed that *Lactobacillus casei* can serve as an effective nutrient regulator for OA treatment by reducing pain, the inflammatory response, and articular cartilage degradation (So et al., 2011). In addition, the GM can regulate many metabolic processes in the host, including energy homeostasis, glucose metabolism, and lipid metabolism (Schoeler and Caesar, 2019). Previous studies of animal models and humans showed that OA is characterized by elevated levels of circulating inflammatory markers, including lipopolysaccharides produced by bacteria, suggesting that proinflammatory metabolites derived from the GM play important roles in OA pathogenesis (Huang et al., 2016; Berthelot et al., 2019). These findings, combined with the correlation between GM dysfunction and OA risk factors, suggest that GM and GM-derived metabolites are involved in OA progression and may be useful as therapeutic targets for OA treatment (Szychlinska et al., 2019).

Miya (MY), a kind of *Clostridium butyricum* tablet, has been reported as a probiotic bacterium used in humans and domestic animals, including as an antidiarrheal in Japan (Seki et al., 2003). Previous studies demonstrated the beneficial properties of MY, including its neuroprotective effects on mood disorders (Tian et al., 2019), improvement of nonalcoholic fatty liver disease induced by a high-fat diet in rats (Seo et al., 2013), inhibition of the cytotoxic effects of *Clostridium difficile in vitro* (Woo et al., 2011), and prevention of gastric ulcers in mice (Wang et al., 2015). MY can improve mucosal damage and inflammation by modifying the abundance of *Bifidobacterium*, *Lactobacillus*, and *Lactococcus*, as well as by altering GM metabolites and promoting the production of anti-inflammatory metabolites by regulating the GM (Hagihara et al., 2018; Hagihara et al., 2020). These findings suggest that MY can maintain intestinal homeostasis by regulating the GM, thus protecting against diseases. In addition, an increasing number of studies has indicated the existence of the gut-muscle axis, suggesting that the gut microbiome affects skeletal muscle health (Liao et al., 2020). However, the effects of MY on OA and its potential role in modulating the gut-muscle-joint axis remain unknown.

Therefore, we constructed a rat OA model to investigate the effects of MY on OA. We also examined the fecal samples of rats using 16S rDNA sequencing and metabolomics analysis to explore the underlying the “gut-muscle-joint” axis mechanisms of MY in OA. Our study provides insights into the treatment of OA with MY.

## Materials and Methods

### Animal Modeling and Grouping

Thirty specific pathogen-free female Wistar rats weighing 180–220 g were purchased from Shanghai SLAC Laboratory Animal Co., Ltd (Shanghai, China). All rats were fed with food and water freely during the experiment and were maintained at 22–25°C and 20–25% humidity, with a 12-h light/dark cycle. After acclimatization for 7 days, all rats were divided into three groups (*n* = 10): control, OA, and OA+MY groups. Rats in the OA and OA + MY groups were used to establish an OA model as described previously (Ouyang et al., 2019). Before modeling, the rats were deeply anesthetized using 2–4% isoflurane and then fixed in the lateral position. The hair of the knee was removed, and a knee incision approximately 4 cm in length was made. The knee capsule was opened laterally; the anterior cruciate ligament was dissected; and the muscles, fascia, and skin were subsequently sutured. After surgery, the rats were intramuscularly injected with penicillin (30,000 U/time) once per day for 3 days. Rats in the control group did not undergo anterior cruciate ligament dissection.

In the second week of modeling, rats in the OA+MY group were administered MY (4 × 10^5^ colony-forming units/mL *C butyricum*, Miyalisan Pharmaceutical Co., Ltd, Nagano, Japan) orally once per day for 4 weeks. Rats in the control and OA groups were treated with an equal volume of phosphate-buffered saline. After treatment for 4 weeks, all rats were killed by cervical dislocation, and knee joint samples, feces samples of rats, and tibia muscle were collected. All animal experiments were conducted in accordance with the National Medical Advisory Committee guidelines, using procedures approved by the Institutional Animal Care and Use Committee.

### Histopathology Analysis

The collected knee joint samples were fixed with 4% paraformaldehyde (China National Pharmaceutical Group Corporation, Shanghai, China) for 24 h at 4°C and decalcified in a 10% EDTA solution at 25°C for approximately 1 month. Knee joint samples were dehydrated using gradient concentrations of alcohol (75% for 2 h, 85% for 2 h, 90% for 1.5 h, 95% for 2 h, and 100% for 2 h). The knee joint samples were washed and embedded in paraffin. The 4 μm sections were cut and stained with hematoxylin and eosin (HE). After dehydration and sealing, the slides were observed under an optical microscope (Olympus, Tokyo, Japan).

### Determination of Succinate Dehydrogenase and Muscle Glycogen

The tibia muscle samples were used to determine the SDH and MG contents using an SDH detection kit (cat. no. A022-1-1, Nanjing Jiancheng Bioengineering Institute, Nanjing, China) and glycogen detection kit (cat. no. A043-1-1, Nanjing Jiancheng Bioengineering Institute) according to the manufacturer’s instructions.

### 16S rDNA Sequencing and Bioinformatics Analysis

The fecal samples from rats (*n* = 10 for each group) in the OA and OA+MY groups were sent to Yanzai Biotechnology (Shanghai) Co., Ltd. (Shanghai, China) for 16S rDNA sequencing on the Illumina MiSeq platform (San Diego, CA, United States). The V3-V4 region of the 16S rRNA gene was amplified using a primer set (341F ACTCCTACGGGAGGCAGCA/806R CGGACTACHVGGGTWTCTAAT), and the amplified products were purified. The sequencing library was constructed using a TruSeq Nano DNA LT Library Prep kit (Illumina) following the manufacturer’s instructions. After quality testing using an Agilent High Sensitivity DNA Kit (Agilent Technologies, Santa Clara, CA, United States) on an Agilent Bioanalyzer, the DNA samples were sequenced on an MisSeq sequencer.

QIIME 2 (2019.4, http://qiime.org/) was used for bioinformatics analysis of the GM. The raw sequencing data were filtered, denoised, and merged, chimeras were removed using DADA2 to obtain the amplicon sequence variants. The Greengenes database (release 13.8) in QIIME2 software was used to assign operational taxonomic units (OTUs) based on the threshold of 97% sequence similarity. Next, the diversity of the GM and differences in the GM between the OA and OA+MY groups at different levels were analyzed. The underlying pathways involved in MY treatment were predicted using the Kyoto Encyclopedia of Genes and Genomes (KEGG) database.

### Metabolite Extraction and Metabolomic Analysis

The fecal samples from rats (*n* = 10 for each group) in the OA and OA+MY groups were dissolved in 1,000 μL of methanol and vortexed for 1 min. After centrifugation at 12,000 rpm for 10 min at 4°C, the supernatant (450 μL) was transferred to a new tube and concentrated to dryness under vacuum. The dried powder was redissolved in 150 μL 2-chlorobenzalanine (4 ppm) in 80% methanol solution and filtered through 0.22 μm membranes; this solution was used for liquid chromatography-mass spectrometry (LC-MS).

LC-MS was performed using an Ultimate 3,000 system (Thermo Fisher Scientific, Waltham, MA, United States) equipped with an ACQUITY UPLC^®^ HSS T3 column (1.8 µm, 150 × 2.1 mm, Waters, Milford, MA, United States) and a Thermo Q Exactive mass spectrometer. The mobile phases were 0.1% formic acid in water (C) and 0.1% formic acid (D) in acetonitrile as the positive control, and 5 mM ammonium formate in water (A) and acetonitrile (B) as the negative control. The elution gradient was set as follows: 0–1 min, 2% B/D; 1–9 min, 2–50% B/D; 9–12 min, 50–98% B/D; 12–13.5 min, 98% B/D; 13.5–14 min, 98–2% B/D; 14–20 min, 2% D for positive or 14–17 min, 2% B for negative. The flow rate was 0.25 ml/min, and the injection volume was 2 μL. The spray voltages of Q Exactive mass spectrometry for positive and negative mode were 3.5 and 2.5 kV, respectively. Full scanning was performed with a resolution of 70,000 and scanning range of 81–1,000 m/z.

The raw data were converted to mzXML format using ProteoWizard software (version 3.0.8789) and then processed using R (version 3.3.2) for peak identification, peak filtration, and peak alignment. The processing results generated a data matrix consisting of the mass-to-charge ratio (m/z), retention time, and peak intensity. Metabolites were identified using an in-house MS2 database based on the RT, m/z, and peak intensity. Differential metabolites were screened according to the thresholds of *p*-value ≤0.05 and VIP ≥1. KEGG database was used to analyze the differential metabolite-linked metabolic pathways.

### Combined Analysis of 16S rDNA Sequencing Data and Metabolomics Data

The correlation between the GM and differential metabolites was calculated using Pearson correlation analysis and redundancy analysis (RDA); *p* < 0.05 was considered statistically significant.

### Real-Time Quantitative PCR

Total RNA was extracted from the tibia muscle samples in different groups using an RNAiso Plus kit (Trizol, Takara, Shiga, Japan) according to the manufacturer’s protocols. The purity and concentration of total RNA was measured using a microplate reader (Thermo Fisher Scientific). The total RNA was reverse-transcribed into cDNA using a PrimeScript™ II 1st Strand cDNA synthesis kit (Takara) according to the manufacturer’s recommendations. The total sample volume used for quantitative reverse transcription polymerase chain reaction (RT-qPCR) was 20 μL, which contained 10 μL SYBR Premix EX Taq (2×), 1 μL forward primer (10 μM), 1 μL reverse primer (10 μM), 2 µL cDNA, and 6 µL distilled water. RT-qPCR was initiated at 50°C for 2 min, 95°C for 2 min, followed by 40 cycles at 95°C for 15 s, 60°C for 60 s, 95°C for 15 s, 60°C for 60 s, and 95°C for 15 s. The sequences of all primers are shown in Table 1, and *GAPDH* served as the normalization control. The relative mRNA levels of related genes were calculated using the 2^−ΔΔCt^ method (Xu et al., 2019).

**TABLE 1:** Sequences of all primers.

Primer	Sequence (5′-3′)
AMPK	rF: CAG​CGA​TCA​ACA​GGC​GAG​AC
	rR: AGA​GAT​ATC​CCA​GCA​AAC​CTA​TCC​A
Tfam	rF: AGA​GTT​GTC​ATT​GGG​ATT​GG
	rR: CAT​TCA​GTG​GGC​AGA​AGT​C
Murf1	rF: ACC​TGC​TGG​TGG​AGA​ACA​TC
	rR: CTT​CGT​GTT​CCT​TGC​ACA​TC
Myod	rF: CGA​CTG​CCT​GTC​CAG​CAT​AG
	rR: GGA​CAC​TGA​GGG​GTG​GAG​TC
Ldh	rF: GCA​GCA​GGG​TTT​CTA​TGG​AG
	rR: TGG​AGA​CAG​TGG​GAT​TGT​CA
Lcad	rF: GCA​GTT​ACT​TGG​GAA​GAG​CAA
	rR: GGC​ATG​ACA​ATA​TCT​GAA​TGG​A
Mcad	rF: CCA​CAG​TGA​CCC​TTT​CTA​G
	rR: GTG​ACA​GGC​TAC​CTT​TCT​T
Chrna1	rF: GGC​ACT​TGG​ACC​TAT​GAC​GGC​TCT
	rR: GAC​GCT​GCA​TGA​CGA​AGT​GGT​AGG
Chrnd	rF: GCC​GCA​AGC​CGC​TCT​TCT​ACA​TCA
	rR: CGT​GCT​GGG​TGT​TCG​GAA​GTG​GAT
Rapsyn	rF: GCT​GAA​GAG​GTT​GGA​AAT​AAG​C
	rR: TCA​CCA​CAG​AGG​CCA​CAG​TAG​A
Agrin	rF: AGA​AGA​ATG​CTT​GCC​CTG​CTA​CG
	rR: ATGCGCCGTTGCTGGTTG
IL1β	rF: CCC​TGC​AGC​TGG​AGA​GTG​TGG
	rR: TGT​GCT​CTG​CTT​GAG​AGG​TGC​T
GAPDH	rF: AGA​CAG​CCG​CAT​CTT​CTT​GT
	rR: CTT​GCC​GTG​GGT​AGA​GTC​AT

### Western Blotting

Total protein was isolated from the tibia muscle samples of the different groups using RIPA lysis buffer (Beyotime Biotechnology, Beijing, China) following the manufacturer’s instructions, and the concentrations of total protein samples were examined using a BCA assay kit (Thermo Fisher Scientific). The total protein samples (20 μg) were separated by 10% sodium dodecyl sulfate-polyacrylamide gel electrophoresis, transferred to polyvinylidene fluoride membranes, and blocked with 5% skim milk at 37°C for 1 h. After washing three times with 1 × buffer composed of 1 ml Tween-20 in 1 L 1× phosphate-buffered saline, the membranes were incubated with anti-AMPK antibody (1:1,000, Cell Signaling Technology, Danvers, MA, United States), anti-Chrna1 antibody (1:1,000, Proteintech, Rosemont, IL, United States), anti-Ldh antibody (1:1,000, Abcam, Cambridge, United Kingdom), anti-Mcad antibody (1:1,000, Proteintech), anti-Myod antibody (1:1,000, Proteintech), anti-Tfam antibody (1:1,000, Proteintech), and anti-GAPDH antibody (1:10,000, Proteintech) at 4°C overnight. After washing with PBST, the membranes were incubated with secondary antibody (1:5,000, Jackson ImmunoResearch, West Grove, PA, United States) at 37°C for 2 h. After washing five times, the protein bands were visualized using a Millipore ECL system (Billerica, MA, United States).

### Statistical Analysis

GraphPad Prism 5 software (GraphPad, Inc., San Diego, CA, United States) was used for statistical analysis, and data were reported as the mean ± standard deviation. One-way analysis of variance followed by the Bonferroni method was used to compare significant difference among more than two groups; student’s *t* test was used to compare two groups. *p* < 0.05 was considered statistically significant.

## Results

### Effects of MY on OA

**FIGURE 1:**
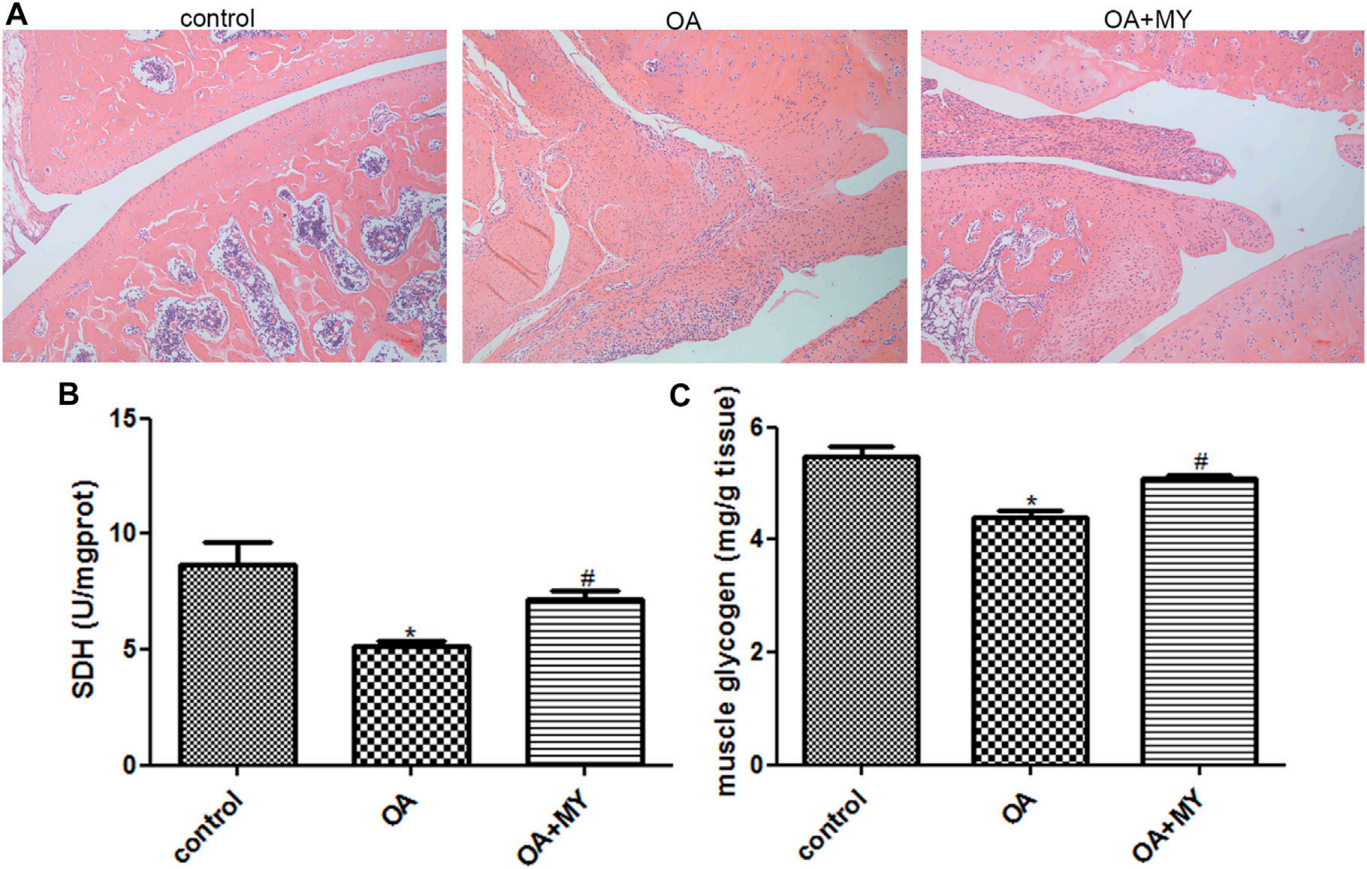
Effects of Miya (MY) on the symptoms and succinate dehydrogenase (SDH) and muscle glycogen (MG) contents in osteoarthritis (OA). **(A)** Histological changes associated with OA and MY treatment in rats were investigated by hematoxylin-eosin staining under a microscope at ×100 magnification. **(B)** Contents of SDH in the tibia muscle of different groups. **(C)** Contents of MG in the tibia muscle of different groups. *p < 0.05, compared with the control group. ^#^ *p* < 0.05, compared with the OA group.

Histological changes associated with OA and MY treatment in rats were investigated using HE staining. As shown in Figure 1A, in rats in the control group, the synovial tissue cells were arranged in an orderly manner, and the chondrocytes were more numerous, round or nearly round in shape, and evenly distributed in each layer of the cartilage. The cell nuclei were clearly visible and uniformly stained. However, rats in the OA group showed hyperplasia and disordered synovial tissue cells; the number of chondrocytes was reduced, cartilage tissue structure was not clearly distinguished, and nuclear staining was shallow. The number of chondrocytes was larger in the OA+MY group than that in the OA group, and the arrangement of the cartilage tissue was more orderly than that in the OA group (Figure 1A). These results indicate that MY treatment can alleviate the symptoms of OA.

### Effects of MY on SDH and MG Contents in OA

The SDH and MG contents in the tibial muscle were determined. The contents of SDH in the control, OA, and OA+MY groups were 8.67 ± 0.98, 5.12 ± 0.28, and 7.19 ± 0.33 U/mgprot, respectively. The SDH content was significantly lower in the OA group than in the control group (*p* < 0.05), whereas it was evidently higher in the OA+MY group than in the OA group (*p* < 0.05, Figure 1B). The contents of the MG in the control, OA, and OA+MY groups were 5.48 ± 0.3, 4.38 ± 0.24, and 5.09 ± 0.12 mg/g tissue, respectively, indicating that the MG and SDH contents in different groups were similar (Figure 1C).

### 16S rDNA Sequencing Analysis

**FIGURE 2:**
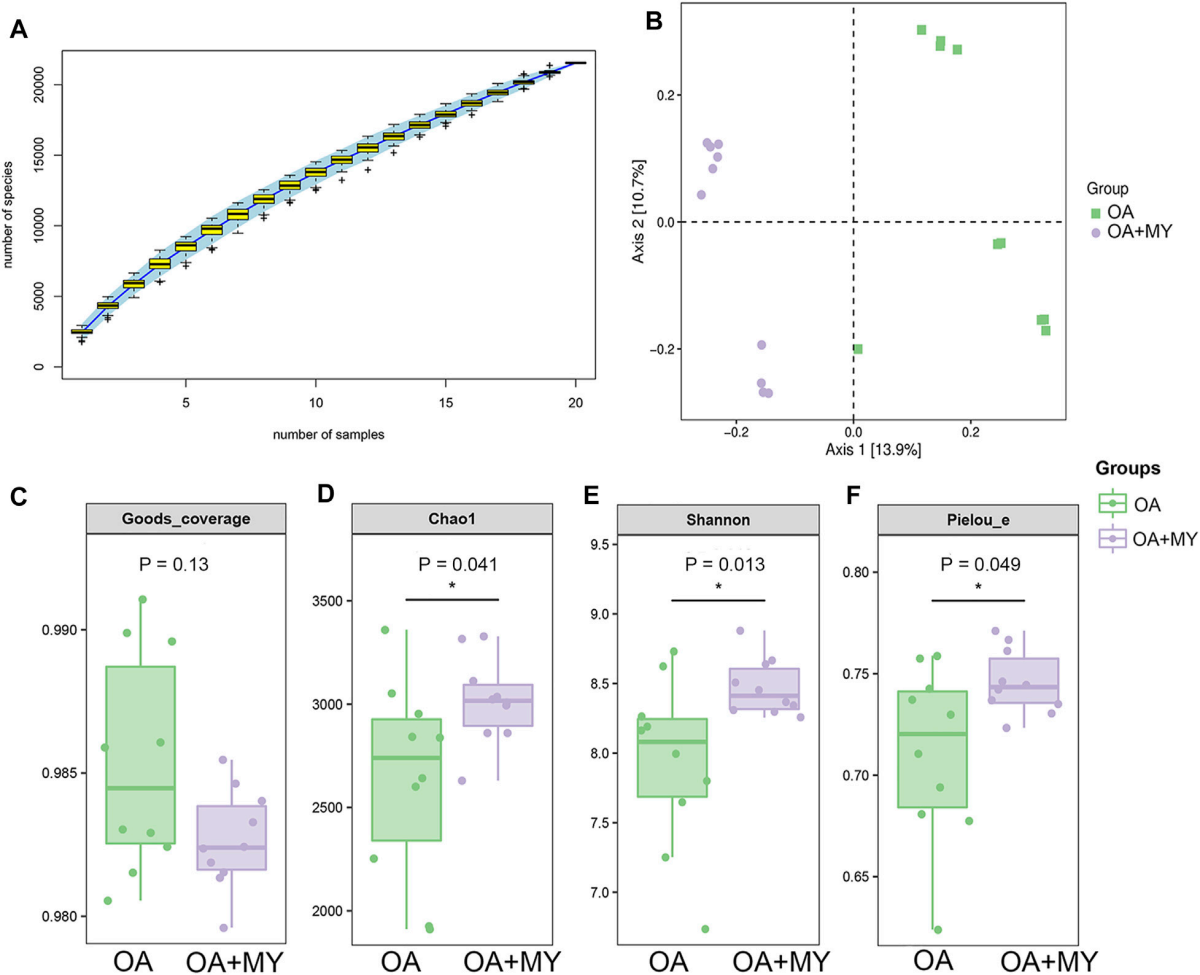
Effects of MY on the overall diversity of gut microbiota in OA. **(A)** Species accumulation curves analysis. **(B)** Beta diversity analysis using pincipal coordinate analysis (PCoA) based on the Jaccard distance. Alpha diversity analyses based on the Goods-coverage **(C)**, Chao1 index **(D)**, and Shannon index **(E)**, and Pielou’s evenness **(F)**. *p < 0.05, compared with the OA group.

The feces of rats in the OA and OA + MY groups were used for 16S rDNA sequencing. The species accumulation curve reached a stable value (Figure 2A), indicating that the depth of sequencing covered rare new phylotypes and captured the greatest diversity. Principal coordinate analysis showed obvious clustering of the microbiota composition in the OA and OA+MY groups, indicating a higher depth and reliability of the sequencing results (Figure 2B). Good’s coverage in the OA and OA+MY groups was 0.985 ± 0.004 and 0.983 ± 0.002, respectively, indicating that the sequencing results covered most species (Figure 2C). The Chao 1, Shannon, and Pielou evenness indices were significantly higher in the OA+MY group than those in the OA group (*p* < 0.05, Figures 2D–F). Together, these results suggest that MY can increase the GM biodiversity that was reduced by OA.

**FIGURE 3:**
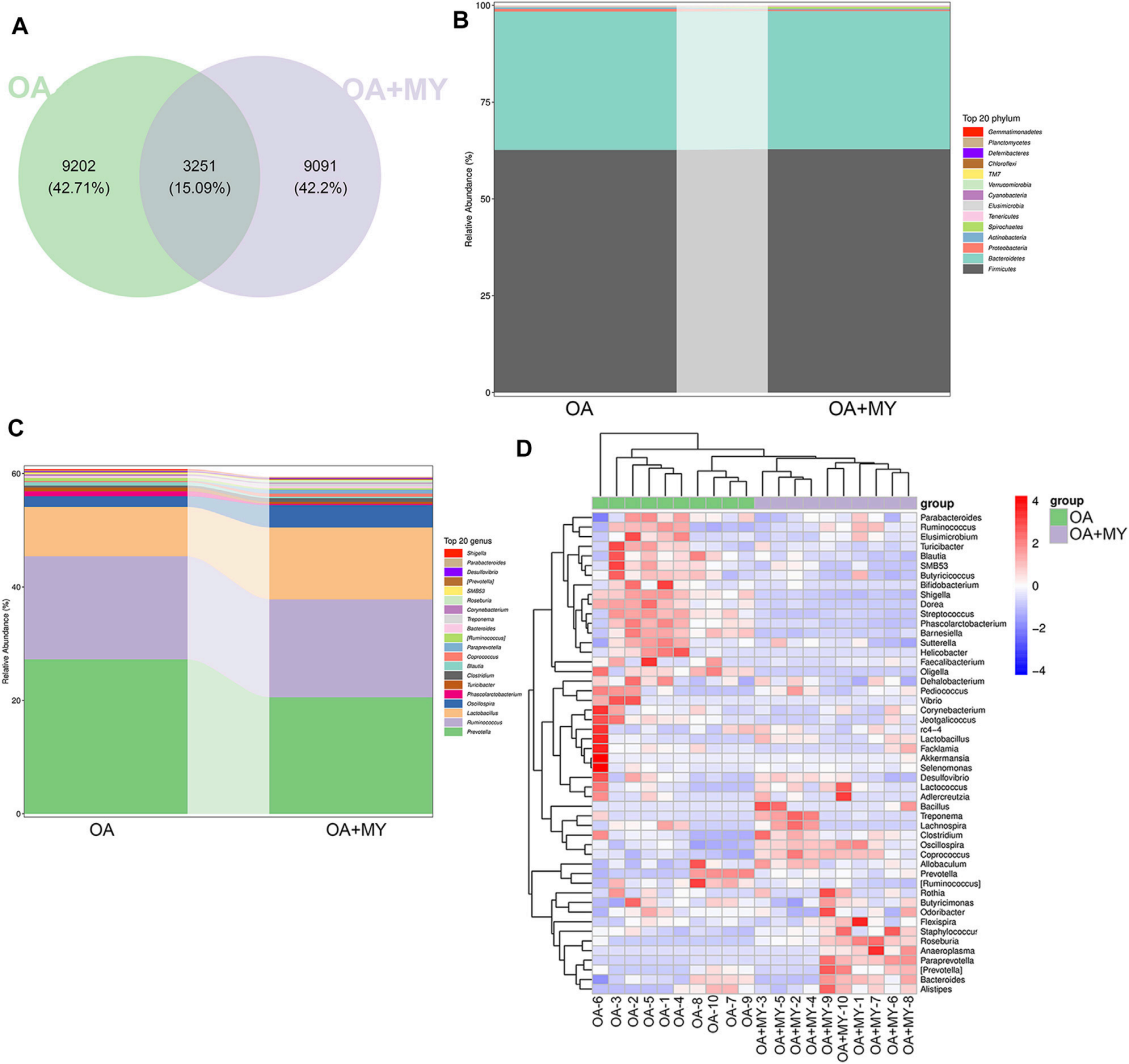
Effects of MY on the gut microbial composition in OA. **(A)** Venn diagrams of operational taxonomic units (OTUs) abundance in the OA and OA+MY groups. **(B)** R abundance of top 20 phyla between the OA and OA+MY groups. **(C)** Relative abundance of top 20 genera between the OA and OA+MY groups. **(D)** Heat map of prominent OTUs (top50) assigned to the genus level among each sample.

**FIGURE 4:**
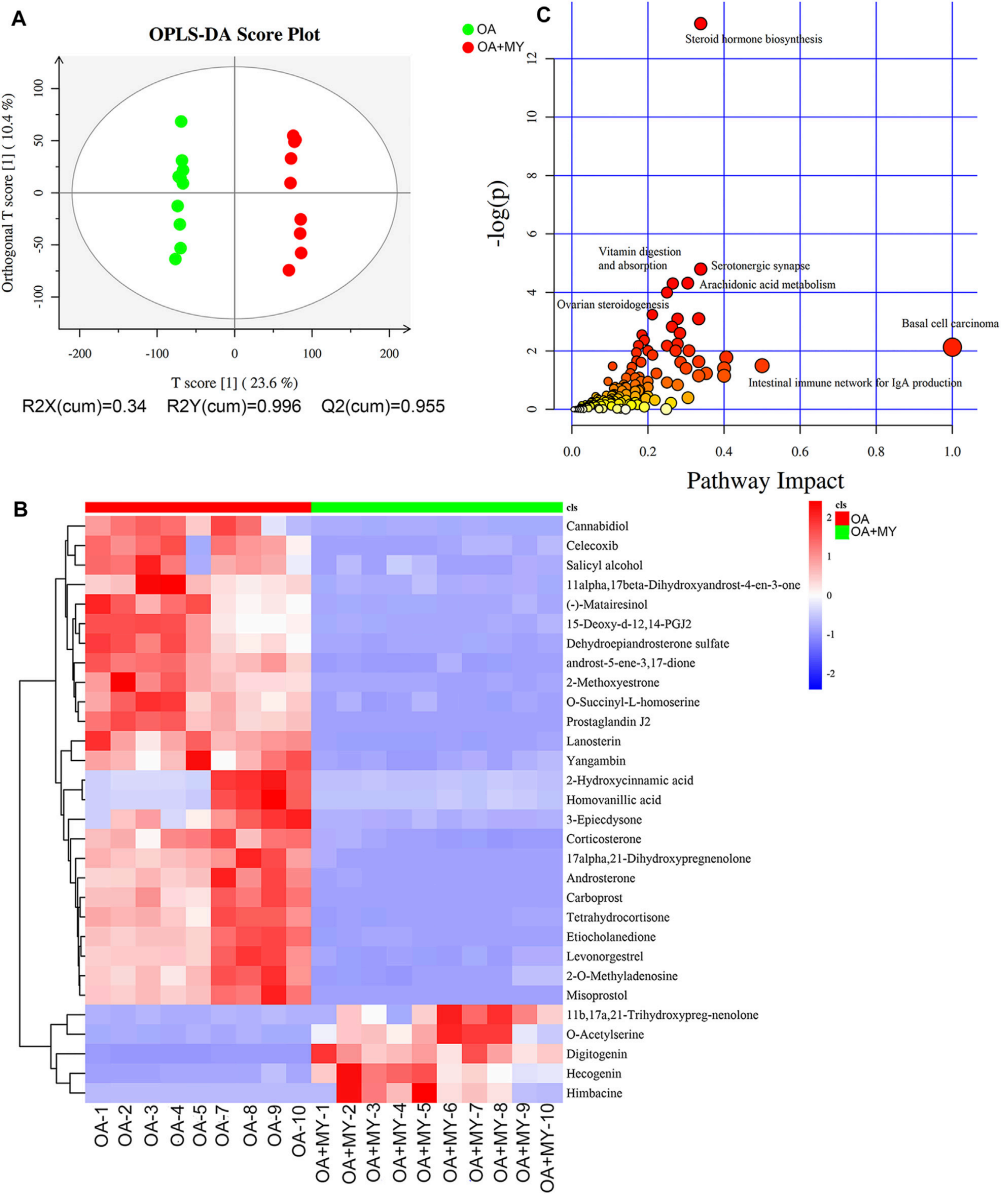
Effects of MY on the fecal metabolomic profiles in OA. **(A)** Orthogonal projections to latent structures discriminant analysis (OPLS-DA) based on the fecal compounds data. **(B)** Agglomerate hierarchical clustering analysis of differential metabolites (Top30). **(C)** Kyoto Encyclopedia of Genes and Genomes pathways map of identified differential metabolites.

We then analyzed the GM composition at the phylum and genus levels. The Venn diagram contained 12,453 and 12,342 OTUs in the OA and OA + MY groups, respectively, including 3,251 shared OTUs (Figure 3A). At the phylum level, Firmicutes, Bacteroidetes, Proteobacteria, and Actinobacteria were the dominant phyla in the OA and OA + MY groups (Figure 3B and Supplementary Table S1). At the genus level, the relative abundances of *Prevotella*, *Ruminococcus*, *Phascolarctobacterium*, and *Turicibacter* were reduced in the OA+MY group compared to those in the OA group, whereas the relative abundances of *Lactobacillus*, *Oscillospira*, *Clostridium*, *Coprococcus*, and *Paraprevotella* were increased in the OA+MY group (Figure 3C and Supplementary Table S2). The clustering heatmap of the differential species between the two groups (Figure 4) showed that the abundance of *Prevotella*, *Desulfovibrio*, *Shigella*, *Helicobacter*, and *Streptococcus* was higher in the OA group than that in the OA+MY group; however, after MY treatment, *Alistipes*, *Lactococcus*, *Paraprevotella*, *Lactobacillus*, *Oscillospira*, *Clostridium*, and *Coprococcus* were enriched (Figure 3D and Supplementary Figure S1).

The differential GM between the OA and OA+MY groups were subjected to functional analysis, and 44 differential metabolic pathways were screened with an adjusted *p* < 0.05 (Supplementary Figure S2). The functions of L-arginine degradation, enteribactin biosynthesis, glyoxylate cycle, polymyxin resistance, NAD salvage pathway, fucose degradation, anhydromuropeptides recycling, and TCA cycle Ⅷ (*Helicobacter*) were suppressed after MY treatment, whereas the pathways of gallate degradation, 3-phenylpropanoate and 3-(3-hydroxyphenyl) propanoate degradation to 2-oxopent-4-enoate, and cinnamate and 3-hydroxycinnamate degradation to 2-oxopent-4-enoate were activated (Supplementary Figure S2).

### Metabolomic Analysis

We further performed metabonomic analysis of MY-treated OA rats. The orthogonal projections to latent structures discriminant analysis model of samples showed that the metabolites significantly differed in the OA and OA+MY groups, suggesting that the fecal metabolic spectrum was greatly altered by MY administration (Figure 4A). Based on the criteria of *p* ≤ 0.05, and variable importance of projection ≥1, 395 differential metabolites were identified, including 204 and 191 down- and upregulated metabolites, respectively (Supplementary Table S3). A clustering heatmap of the top 30 differential metabolites is shown in Figure 4B. The relative concentrations of salicyl alcohol, *o*-succinyl-L-homoserine, prostaglandin J2, homovanillic acid, corticosterone, tetrahydrocortisone, and misoprostol were reduced in the OA+MY group, whereas the relative concentrations of 11b,17a, 21-trihydroxypreg-nenolone, *o*-acetylserine, digitogenin, and himbacine were increased (Figure 4B). Further metabolic pathway enrichment analysis indicated that MY mainly affected basal cell carcinoma, the intestinal immune network for IgA production, steroid hormone biosynthesis, vitamin digestion and absorption, serotonergic synapses, arachidonic acid metabolism, and ovarian steroidogenesis (Figure 4C).

### Combined Analysis of 16S rDNA Sequencing and Metabolomics

**FIGURE 5:**
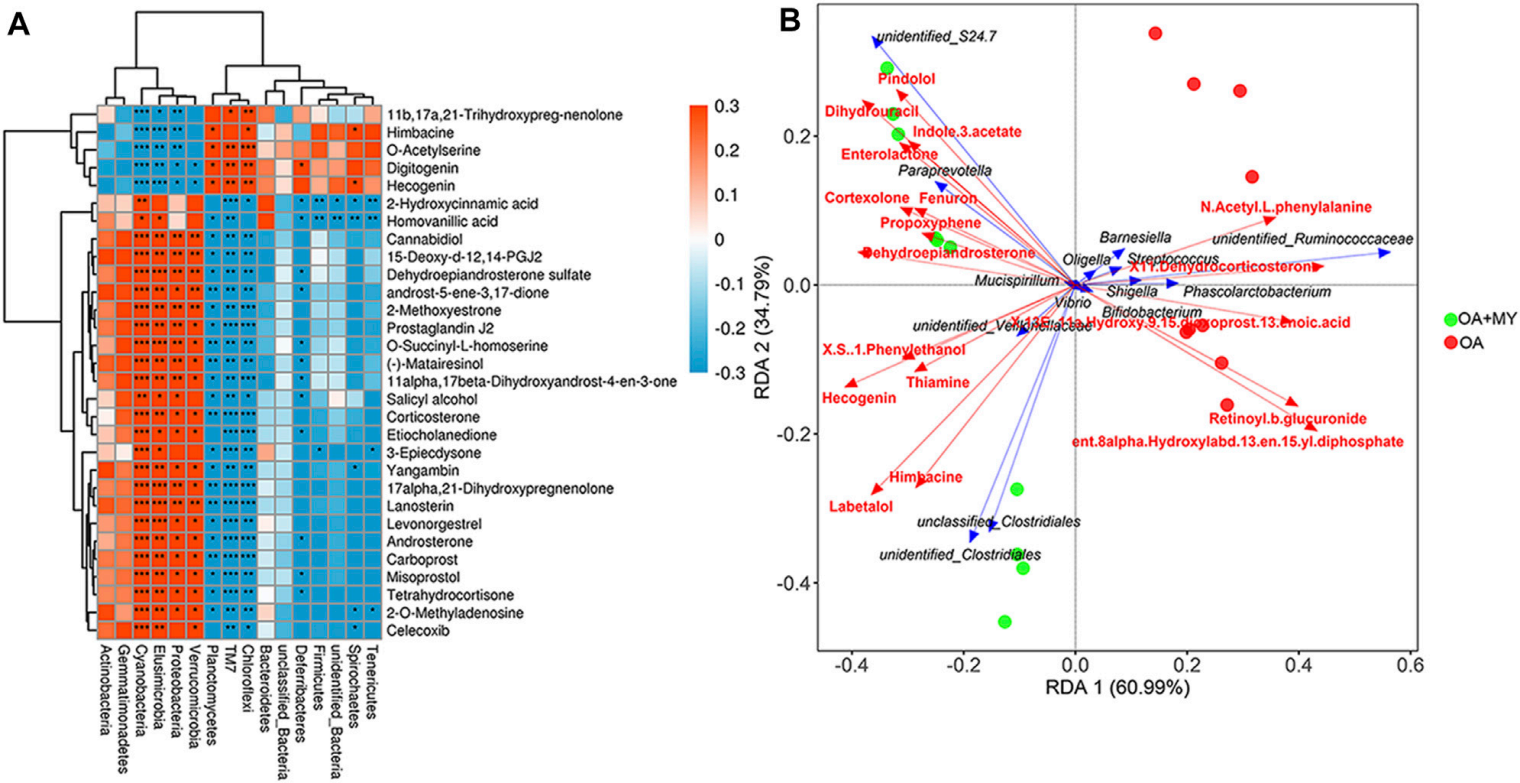
Integrative analysis of 16S rDNA sequencing and metabolomics. **(A)** Associated heat map between the fecal bacteria at the phylum level and top 30 differential metabolites. Different colors and depth represent the size of the correlation coefficient. Red and blue represent positive and negative correlations, respectively. A darker color indicates a higher the correlation. *: 0.01 < *p* < 0.05; **: 0.0001 < *p* < 0.01; ***: *p* < 0.0001. **(B)** Redundancy analysis (RDA) of specific gut microbiota at the genus level responding to metabolites. An acute angle between two variables represents a positive correlation, that is, a synergistic effect. An obtuse angle between two variables indicates a negative correlation, that is, an antagonistic effect.

To investigate the relationship between the GM structure and differential metabolites under altered physiological states, Pearson correlation coefficients were determined and redundancy analysis was performed at the phylum and genus levels. At the phylum level, Actinobacteria and Proteobacteria were negatively correlated with 11b,17a, 21-trihydroxypreg-nenolone, himbacine, o-acetylserine, digitogenin, and hecogenin and positively correlated with o-succinyl-L-homoserine, homovanillic acid, and 2-hydroxycinnamic acid (Figure 5A). In contrast, the relationships between Firmicutes as well as Bacteroidetes and significantly differential metabolites were opposite to those between the former two phyla and metabolites (Figure 5A). The associated heat map between fecal bacteria at the genus level and identified differential metabolites are shown in Supplementary Figure S3. Additionally, RAD was used to analyze the correlation between some specific GM at the genus level and significant metabolites (Figure 5B). For example, *Streptococcus* had synergistic effects with N-acetyl-L-phenylalanine, X11-denydrocorticosteron, and retinoyl-b-glucuronide, whereas it had antagonistic effects with pindolol, thiamine, and hecogenin. Furthermore, Clostridiales had synergetic effects with retinoyl-b-glucuronide, himbacine, and labetalol but antagonistic actions with N-acetyl-L-phenylalanine, indole-3-acetate, and fenuron (Figure 5B).

### RT-qPCR and Western Blot Analysis

**FIGURE 6:**
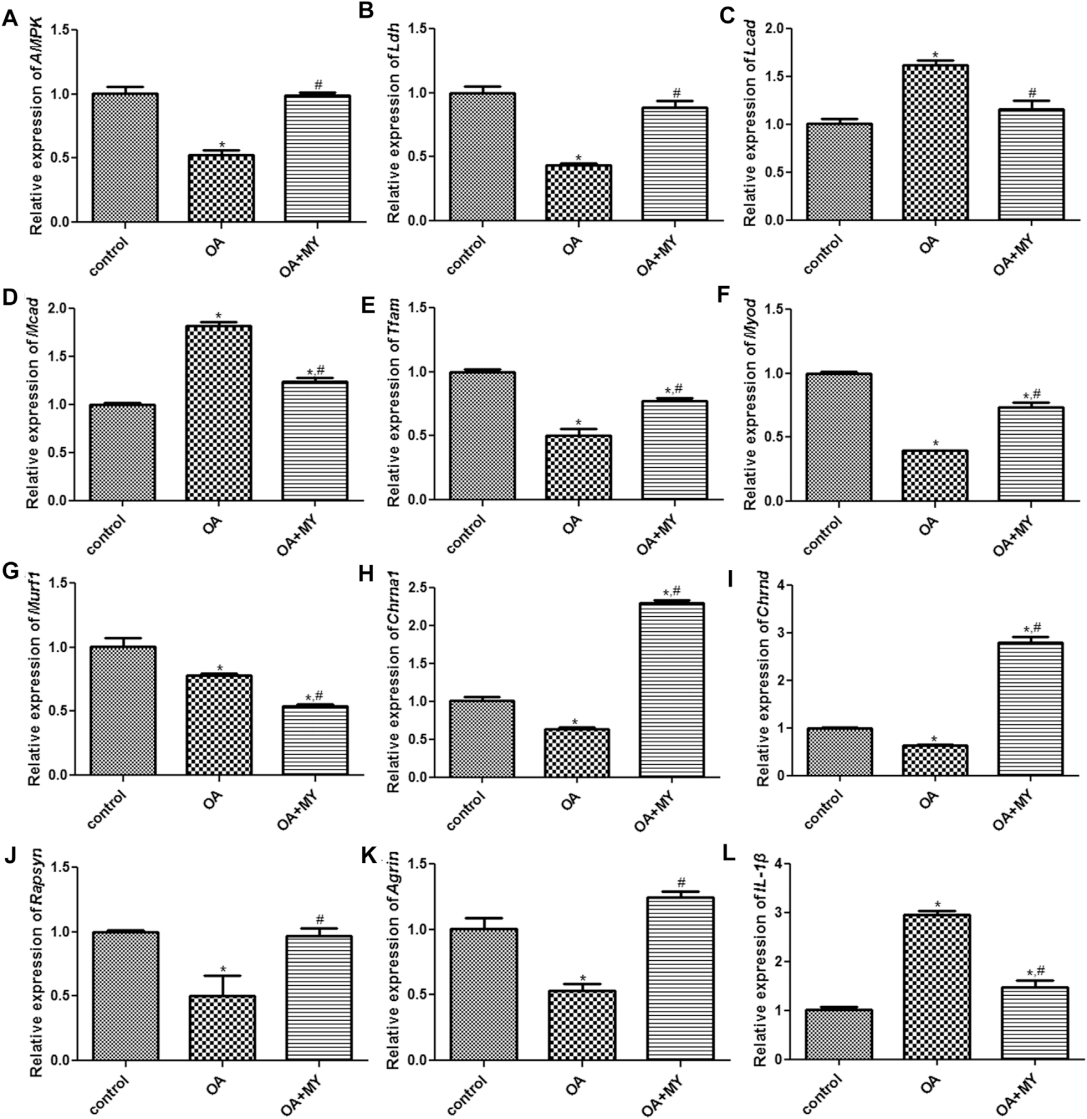
Effects of MY on the expressions of related genes in the tibia muscle of different groups. mRNA expression of *AMPK* **(A)**, *Ldh* **(B)**, *Lcad* **(C)**, *Mcad* **(D)**, *Tfam* **(E)**, *Myod* **(F)**, *Murf1* **(G)**, *Chrna1* **(H)**, *Chrnd* **(I)**, *Rapsyn* **(J)**, *Agrin* **(K)**, and *IL-1β* **(L)**. *p < 0.05, compared with the control group; ^#^ *p* < 0.05, compared with the OA group.

**FIGURE 7:**
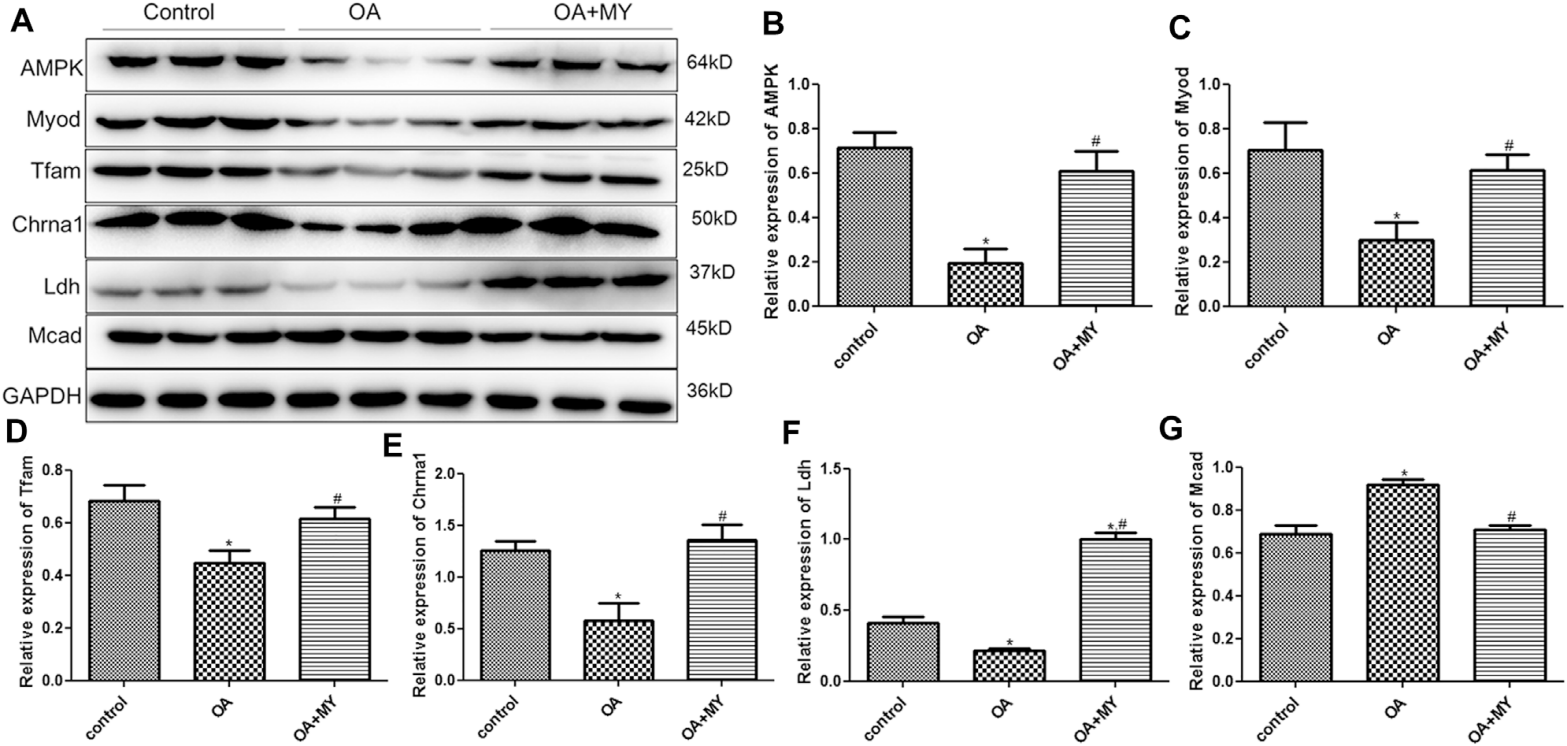
Effects of MY on the expressions of related proteins in the tibia muscle of different groups. **(A)** Protein bands visualized by western blotting. Protein expression of AMPK **(B)**, Myod **(C)**, Tfam **(D)**, Chrna1 **(E)**, Ldh **(F)**, and Mcad **(G)**. *p < 0.05, compared with the control group; ^#^ *p* < 0.05, compared with the OA group.

Finally, the expression of energy metabolism-related genes (*AMPK*, *Ldh*, *Lcad*, *Mcad*, *Tfam*), myogenesis-associated genes (*Myod* and *Murf1*), neuromuscular junction (NMJ)-related genes (*Chrna1, Chrnd*, *Rapsyn* and *Agrin*), and inflammatory cytokines (*IL-1β*) in the tibia of different groups were determined using RT-qPCR and western blotting. The mRNA expression of *AMPK*, *Tfam*, and *Ldh* was significantly downregulated in the OA group compared to that in the control group (*p* < 0.05), and MY significantly upregulated their expression levels compared to that in the OA group (*p* < 0.05, Figures 6A,B,E). However, the trend in the mRNA expression of *Lcad* and *Mcad* in the different groups was opposite to that of *AMPK*, *Ldh*, and *Tfam* (Figures 6C,D). The mRNA expression of myogenesis-associated genes (*Myod*, *Murf1*) was significantly downregulated in the OA group compared to that in the control group (*p* < 0.05), whereas MY significantly upregulated *Myod* mRNA expression and further significantly downregulated *Murf1* expression (*p* < 0.05, Figures 6F,G). For NMJ-related genes, the mRNA expression levels of *Chrna1* and *Chrnd* were significantly lower in the OA group than in the control group (*p* < 0.05), whereas they were significantly higher after MY administration (*p* < 0.05, Figures 6H,I). The mRNA expression levels of *Rapsyn* and *Agrin* in the different groups were similar to those of *AMPK* (Figures 6J,K). The mRNA expression of IL-1β was evidently upregulated in the OA group compared to that in the control group (*p* < 0.05), and MY treatment markedly downregulated its level that was increased in OA (*p* < 0.05, Figure 6L). Western blotting was performed to measure the protein expression levels of AMPK, Myod, Tfam, Chrna1, Ldh, and Mcad (Figure 7A); the results for protein expression levels in the different groups detected agreed with those obtained after RT-qPCR (Figures 7B–G).

## Discussion

OA is common worldwide and a major cause of disability that negatively influences people’s physical and mental health. MY is a product of gram-positive obligate anaerobes that can promote health by regulating the GM and intestinal homeostasis (Hagihara et al., 2020). We established a rat OA model and treated the rats with MY. It was found that MY treatment could promote joint repair and improve the symptoms of OA. In order to understand the effects of GM and GM-derived metabolites on OA, 16S rDNA sequencing and metabolomic analyses were performed. MY significantly increased the values of Chao1, Shannon and Pielou’s evenness compared with the OA group, which indicated that MY could enhance the biodiversity caused by OA. A previous study reported that high-fat diets contribute to severe cartilage degeneration and decrease the diversity of the GM in mice, and that wheel running exercise relieved cartilage degeneration and increased GM diversity induced by high-fat diets (Li et al., 2021). Chen et al. also found that the abundance and diversity of the GM were reduced in OA, and moxibustion showed a favorable regulatory effect on the GM (Chen et al., 2020). These results suggest that MY can improve intestinal health in patients with OA by increasing GM diversity.

The GM composition was analyzed at the phylum and genus levels. The dominant phyla in the OA and OA+MY groups were Firmicutes, Bacteroidetes, Proteobacteria, and Actinobacteria, with no significant differences in their abundances between the two groups. At the genus level, the abundances of *Prevotella*, *Ruminococcus*, *Desulfovibrio*, *Shigella*, *Helicobacter*, and *Streptococcus* were higher in the OA group, whereas *Lactobacillus*, *Oscillospira*, *Clostridium*, and *Coprococcus* were enriched after MY treatment. Additionally, these differential GM were enriched in 44 significantly different metabolic pathways. OA is closely associated with metabolic disorders and low-grade systemic inflammation. An increased abundance of *Prevotella* can enhance T-helper cell 17-mediated mucosal inflammation, which affects rheumatoid arthritis, metabolic disorders, periodontitis, and low-grade systemic inflammation (Larsen, 2017). *Ruminococcus* generates inflammatory polysaccharides and is involved in inflammation (Henke et al., 2019). *Desulfovibrio* is an endotoxin-producing bacterium (Li et al., 2021) with an increased abundance in patients with symptomatic hand OA (Wei et al., 2021). *Shigella*, *Helicobacter*, and *Streptococcus* are harmful bacteria, and increases in their abundance can lead to increased pain in the knee joint (Boer et al., 2019; Honcharuk et al., 2021). *Lactobacillus*, a type of probiotic, has been reported to alleviate pain and reduce cartilage degradation in OA rats by inhibiting proinflammatory cytokines (Lee et al., 2018). The bacterium *Oscillospira* is not well understood but is positively correlated with weight loss and health (Konikoff and Gophna, 2016), and *Coprococcus* and *Clostridium butyricum* are butyrate-producing probiotics that can reduce systemic inflammation (Zhao et al., 2020). Combined with our results, MY may enhance joint repair and improve OA by increasing the abundance of beneficial bacteria (*Lactobacillus*, *Oscillospira*, *Clostridium*, and *Coprococcus*) and decreasing the abundance of pathogenic microorganism (*Prevotella*, *Ruminococcus*, *Desulfovibrio*, *Shigella*, *Helicobacter*, and *Streptococcus*).

Through metabolomic analysis, a total of 395 differential metabolites were identified, including 204 downregulated metabolites (*o*-succinyl-l-homoserine, corticosterone, and homovanillic acid) and 191 upregulated metabolites (*o*-acetylserine and digitogenin). Functional analysis showed that these differential metabolites were enriched in the intestinal immune network for IgA production, steroid hormone biosynthesis, vitamin digestion and absorption, and arachidonic acid metabolism. IgA is the primary form of mucosal surface-secreted antibodies and plays a key role in maintaining intestinal homeostasis (Zhao and Elson, 2018). A previous bioinformatics study identified 174 differentially expressed genes between synovial membranes with and without inflammation and suggested that these genes function through the intestinal immune network for IgA production (Lin et al., 2018). Xia et al. (Xia et al., 2019) showed that Rehmanniae Radix Preparata prevented dexamethasone-induced bone loss, mainly by interfering with steroid hormone biosynthesis. Vitamins such as vitamins K, D, and E are associated with OA progression. Previous studies reported that supplementation with these vitamins can prevent and improve OA because of their antioxidant and anti-inflammatory effects (Heidari and Babaei, 2019; Fuggle et al., 2020). Arachidonic acid and its metabolite prostaglandin E2 are key modulators of osteoclast differentiation and play important roles in bone diseases including OA (Abshirini et al., 2021). Therefore, MY may relieve OA by regulating the pathways of the intestinal immune network for IgA production, steroid hormone biosynthesis, vitamin digestion and absorption, and arachidonic acid metabolism. However, these predictions require further evaluation.

Further to explore the correlation between GM composition and differential metabolites, integrative analysis of 16s rDNA sequencing results and metabolomics results was carried out. We found that at the phylum level, Actinobacteria and Proteobacteria were negatively correlated with *o*-acetylserine, digitogenin, and hecogenin and positively correlated with *o*-succinyl-l-homoserine and homovanillic acid; however, the opposite correlations were observed between Firmicutes and Bacteroidetes and these metabolites. At the genus level, RAD analysis showed that Clostridiales had synergetic effects with retinoyl-b-glucuronide and labetalol, whereas they had antagonistic action on N-acetyl-L-phenylalanine and indole-3-acetate and fenuron. *Streptococcus* was positively related to N-acetyl-L-phenylalanine, X11-denydrocorticosteron, and retinoyl-b-glucuronide and negatively associated with pindolol, thiamine, and hecogenin. Taken together, MY may improve joint damage and OA by maintaining GM homeostasis, supporting the existence of a “gut-joint” axis in OA.

Skeletal muscle is the main organ that takes up glucose and oxidizes fatty acids and plays an important role in metabolism. A previous study reported that the GM can contribute to skeletal muscle mass and function in mice, suggesting the presence of a “gut-muscle” axis (Lahiri et al., 2019). Therefore, we examined the function of the tibial muscle. The TCA cycle is the main pathway for cells to obtain energy as well as a common metabolic pathway involved in the complete oxidation of sugars, lipids, and proteins (Thomas et al., 2014). SDH, a respiratory chain enzyme in the TCA cycle, is involved in oxidative phosphorylation and respiratory metabolism (Lan et al., 2019). A previous study showed that elevated superoxide dismutase activity is associated with *Lactobacillus plantarum*, *Lactococcus lactis*, *Lactobacillus fermentae*, and *Lactobacillus gravii* (Spyropoulos et al., 2011). MG participates in supplying energy to the muscle, and disorders of MG levels may lead to loss of muscle strength and function, impairing bioenergetic metabolism (Philp et al., 2012). Germ-free mice were reported to have lower MG levels compared to mice with a normal GM composition (Nay et al., 2019). We found that the SDH and MG levels were significantly reduced in OA mice compared to those in control mice, and MY treatment restored their levels to a level similar to that of control mice. Thus, MY may increase the SDH and MG contents induced by OA in the muscle by regulating the GM to improve joint injury.

In addition, we found that the expression levels of *AMPK*, *Tfam*, *Myod*, *Ldh*, *Chrna1*, *Chrnd*, *Rapsyn*, and *Agrin* were downregulated and those of *Lcad*, *Mcad*, and *IL-1β* were upregulated in the tibial muscle of OA mice, whereas MY significantly reversed the effects of OA. Murf1 expression was significantly downregulated in OA mice, and MY further downregulated its expression. *AMPK*, an intracellular sensor of ATP consumption, controls muscle fiber size by activating the FoxO-mediated protein degradation pathway and is a key regulator of skeletal muscle metabolism (Greer et al., 2007). During OA progression, suppression of the AMPK signaling pathway can inhibit matrix catabolic responses in articular chondrocytes, leading to mitochondrial dysfunction and increased oxidative stress (Yi et al., 2021). *Ldh* is involved in glucose metabolism and serves as a measure of muscle injury (Bazzucchi et al., 2019). *Lcad* and *Mcad* are related to fatty acid oxidation. A previous study by Chang and Kim (Chang and Kim, 2019) showed that vitamin D activates AMPK and upregulates *Lcad*, *Mcad*, and *Tfam* in the skeletal muscle, thus improving muscle fat accumulation and mitochondrial functions. *Tfam* participates in mitochondrial DNA replication, repair, and gene transcription. Overexpression of *Tfam* can enhance energy expenditure and mitochondrial fatty acid oxidative capacity in the skeletal muscle but reduce oxidative stress (Koh et al., 2019). *Myod*, a marker of myogenesis, regulates skeletal muscle differentiation, and its overexpression has been reported to be sufficient to convert non-muscle cells into myoblast-like cells, thereby promoting muscle regeneration (Hernandez-Hernandez et al., 2017). *Murf1*, which encodes E3 ubiquitin ligases, is increased in knee OA and is related to muscle atrophy (Cunha et al., 2019). Interestingly, we found that the expression of Mufr1 was downregulated in OA mice and further declined after MY treatment. This may be related to the complex pathogenesis of OA. Additionally, we only measured the mRNA level of *Murf1*, and further experiments should be conducted to evaluate the protein expression of Murf1 in the muscle.

A previous study indicated that knee OA induces muscle atrophy and NMJ remodeling in the tibialis anterior muscle (Cunha et al., 2019). Choline is a substrate derived from the neurotransmitter acetylcholine and is critical for membrane integrity (Jope and Jenden, 1980). *Chrna1* and *Chrnd*, which encode different acetylcholine subunits and receptors, were reported to influence NMJ development and function (Akaaboune et al., 1999). *Rapsyn* and *Agrin*, NMJ-related genes, are associated with the formation, maturation, and maintenance of the NMJ, and play important roles in the development and assembly of acetylcholine in the NMJ (Lahiri et al., 2019). Our results showed that MY significantly upregulated *Chrna1, Chrnd*, *Rapsyn*, and *Agrin* expression, indicating that MY promotes acetylcholine assembly and NMJ maturation. Furthermore, *IL-1β*, a proinflammatory cytokine, is highly expressed in OA and plays an essential role in cartilage remodeling (Chien et al., 2020). Chien et al. (Chien et al., 2020) indicated that Noggin can prevent cartilage degeneration and inhibit inflammation in OA by reducing the expression of IL-1β and BMP2. Together with our results, MY may regulate energy metabolism-related genes (*AMPK*, *Ldh*, *Lcad*, *Mcad*, and *Tfam*), myogenesis-associated genes (*Myod* and *Murf1*), NMJ-related genes (*Chrna1*, *Chrnd*, *Rapsyn*, and *Agrin*), and inflammatory cytokines (*IL-1β*) in the muscles to suppress OA inflammation and promote joint damage repair, supporting the existence of the “muscle-joint” axis.

In conclusion, MY may promote joint damage repair and protect OA via the “gut-muscle-joint” axis. Specifically, MY may increase the abundance of beneficial bacteria (*Lactobacillus*, *Oscillospira*, *Clostridium*, and *Coprococcus*) and decrease the abundance of pathogenic microorganisms (*Prevotella*, *Ruminococcus*, *Desulfovibrio*, *Shigella*, *Helicobacter*, and *Streptococcus*) in the gut and regulate energy metabolism-related genes, myogenesis-associated genes, NMJ-related genes, and *IL-1β* in the muscles, consequently facilitating joint damage repair and ameliorating OA. This study reveals that the “gut-muscle-joint” axis is involved in OA and provides a theoretical basis for using MY to treat OA.

## Data Availability

The original contributions presented in the study are publicly available. This data can be found here: https://www.ncbi.nlm.nih.gov/bioproject/, PRJNA813999. http://www.ebi.ac.uk/metabolights/MTBLS4457.

## Supplementary Material

Supplementary Figure S1 Effects of MY on the gut microbial composition at genus level. The heat map of the prominent OTUs (top50) assigned to genus level between the OA and OA+MY groups.

Supplementary Figure S2 The functional analysis of differential gut microbiota.

Supplementary Figure S3 The associated heat map between the fecal bacteria at genus level and the identified differential metabolites. Different colors and depth represent the size of the correlation coefficient. Red represents a positive correlation, while blue represents a negative correlation. The darker the color, the higher the correlation. *: 0.01 < P < 0.05; **: 0.0001 < P < 0.01; ***: P < 0.0001.
