# Supplementary material for: Miya Improves Osteoarthritis Characteristics via the Gut-Muscle-Joint Axis According to Multi-Omics Analyses
Source: Front Pharmacol. 2022 May 20;13:816891. doi: 10.3389/fphar.2022.816891 (PMC9163738; doi:10.3389/fphar.2022.816891)
Supplement: Supplementary file 1 [file DataSheet4.pdf]

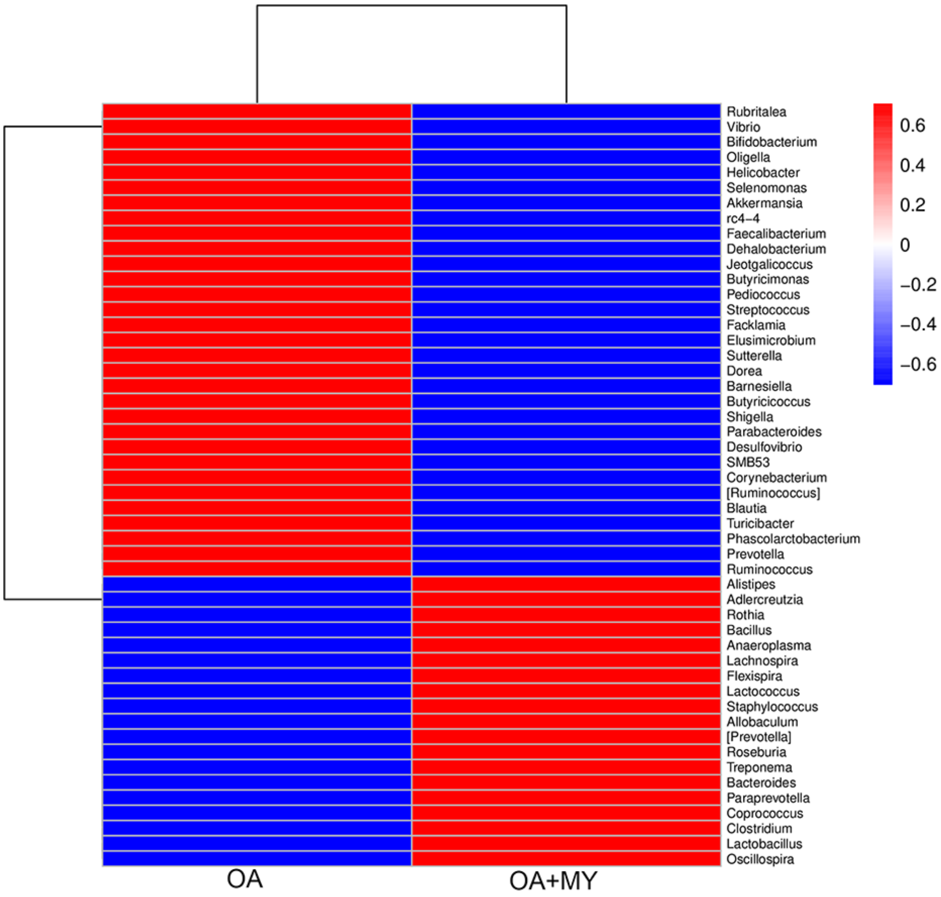

Supplementary table 1 Effects of MY on the gut microbial composition at genus level. The heat map of the prominent OTUs (top50) assigned to genus level between the OA and OA+MY groups.

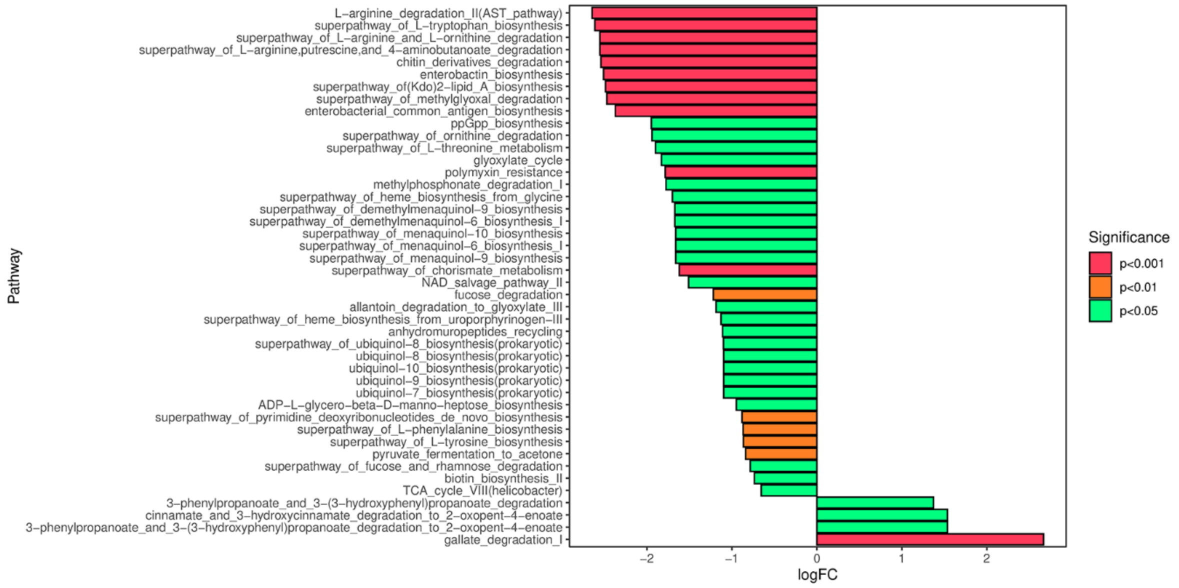

Supplementary Figure 2 The functional analysis of differential gut microbiota.

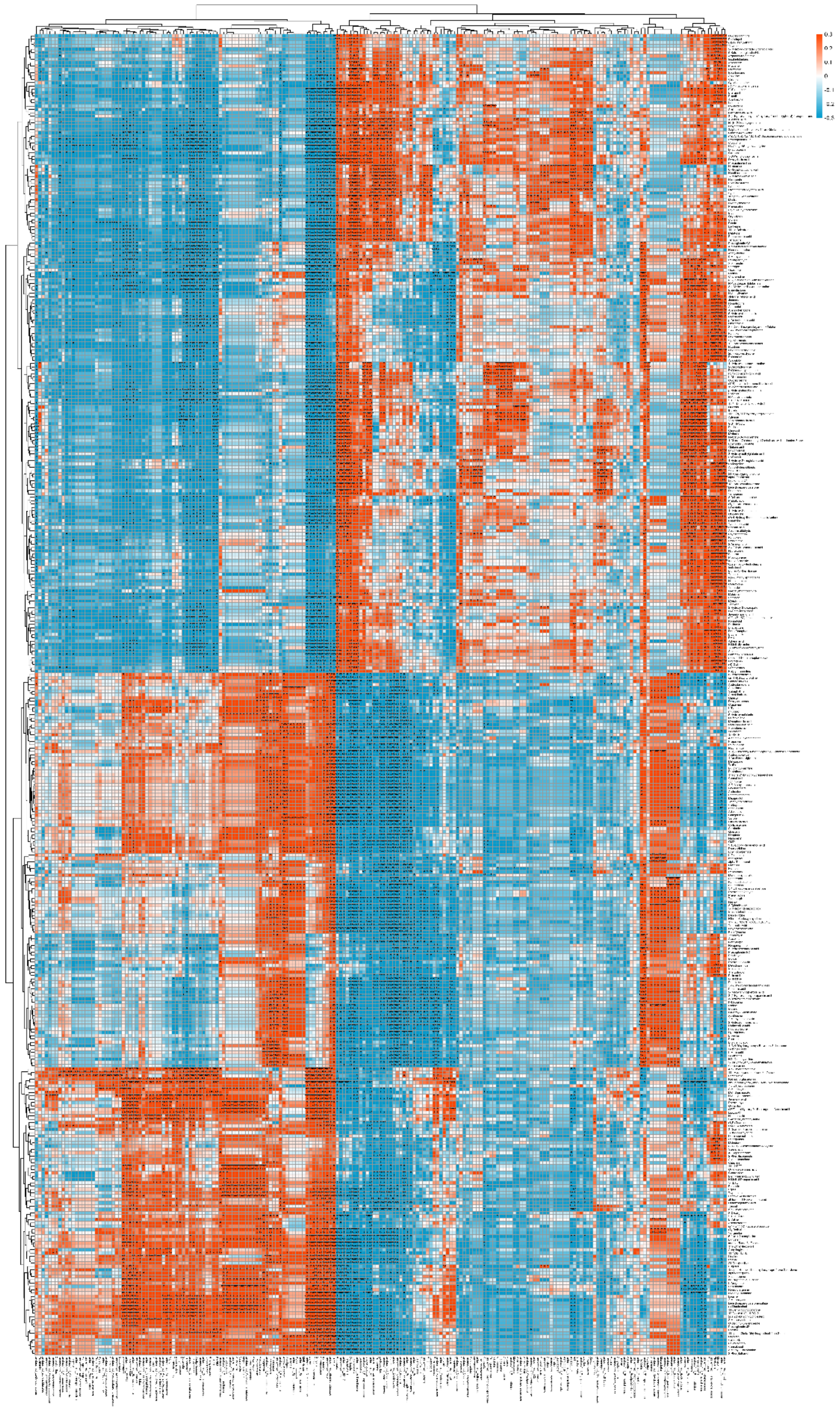

Supplementary Figure 3 The associated heat map between the fecal bacteria at genus level and the identified differential metabolites. Different colors and depth represent the size of the correlation coefficient. Red represents a positive correlation, while blue represents a negative correlation. The darker the color, the higher the correlation. *: 0.01<P < 0.05; **: 0.0001<P< 0.01; ***: P < 0.0001.
